# A maintenance time prediction method considering ergonomics through virtual reality simulation

**DOI:** 10.1186/s40064-016-2886-x

**Published:** 2016-08-02

**Authors:** Dong Zhou, Xin-xin Zhou, Zi-yue Guo, Chuan Lv

**Affiliations:** 1 Beihang University Key Laboratory of Virtual and Reality, Beijing, China; 2 The Reliability and Environment Defense Laboratory, Beijing, China

**Keywords:** Maintainability, Ergonomics, Virtual reality, Motion–time mechanism

## Abstract

Maintenance time is a critical quantitative index in maintainability prediction. An efficient maintenance time measurement methodology plays an important role in early stage of the maintainability design. While traditional way to measure the maintenance time ignores the differences between line production and maintenance action. This paper proposes a corrective MOD method considering several important ergonomics factors to predict the maintenance time. With the help of the DELMIA analysis tools, the influence coefficient of several factors are discussed to correct the MOD value and the designers can measure maintenance time by calculating the sum of the corrective MOD time of each maintenance therbligs. Finally a case study is introduced, by maintaining the virtual prototype of APU motor starter in DELMIA, designer obtains the actual maintenance time by the proposed method, and the result verifies the effectiveness and accuracy of the proposed method.

## Background

Maintainability, as an intrinsic property that shows how well a product can be maintained, should be strictly controlled in the design stage. Maintenance time is a critical quantitative index in maintainability design, which affects the combat mission success rate and cost of the equipment directly. For example, in military fields, short maintenance time will reduce the downtime caused by maintenance, improve the executing ability of equipment to increase combat efficiency, and also in the field of manufacturing, downtime of product line will cause great loss. But there isn’t a way to verify if the maintenance time can meet maintenance requirements in the design stage. Traditional way to measure the maintenance time by the physical prototypes has many drawbacks.

One is that measuring maintenance time by the actual maintenance work relies on physical prototype which is lagged and modifying the design is very difficult in the in the later stage. The other one is that the data used in the cumulative model are usually obtained by the statistical experiment of skilled workers. Convening experienced maintenance personnel is troublesome in the actual work. The development of virtual reality technology provides an effective way to overcome the problem. The application of virtual reality (VR) in maintenance simulation to do analysis and forecasting in the early design phase has been investigated for years. “Virtual maintenance (VM)” is a computer and virtual reality-based application technology that can simulate the maintenance process.

Since the 1990s, many studies have tested VM and proposed a number of solutions. Caudell and Mizell proved the effectiveness of using a VR system to provide instructions for wiring harness assembly (Caudell and Mizell 1992). Real-time immersive virtual environments (VEs), such as the Workbench (Cutler et al. 2010) and the CAVE (Cruzneira et al. 1993) have been used to assess the maintainability of virtual prototypes. Such environments are part of a more complex VR system (Fern et al. 2002) that supports assembly and disassembly operations in immersive VEs. Compared with the object-oriented prototype system called V-REALISM for maintenance training proposed by Qing-Hui and Li (2006), a better solution has been presented by Abate et al. (2009), which combines VR techniques and haptic interaction to simulate process of product assembly maintenance in the aerospace industry. VM systems have been applied in maintenance process simulation (De Sa and Zachmann 1999), maintenance planning (Van Houten and Kimura 2000), and maintenance training (Leino et al. 2009). Christiand et al. (2009) proposed a novel assembly optimization framework based on genetic algorithm; this framework allows the determination of an optimal plan for maintenance processes by following an optimal assembly sequence and by considering factors in path planning. A 3D real-time simulation system for the international thermonuclear experimental reactor (ITER) remote maintenance analysis has been applied to the blanket maintenance manipulator (Esque et al. 2007; Carlo et al. 2009; Cock et al. 2009; Elzendoorn et al. 2009). Amos et al. (2008) have established a machine service support system that demonstrates advanced use of 3D graphical simulation tools in the resource domain, and expends the use of simulation modules from the system design and development phase into operation phase. Bourdot et al. (2008) presented an approach for integrating VR and computer-aided design (CAD), allowing intuitive and direct 3D edition on CAD objects within VEs. More researched models, such as the maintainability evaluation model (Chen and Cai 2003), optimal maintenance policies in incomplete repair models (Kahle 2007), effective visualization model (Tang et al. 2006), and some research have study virtual ergonomics. Sanjog et al. (2012) take the ergonomics into consideration an effort has been made through extensive literature review to highlight relevance of digital human modeling software as a tool for evaluating, improving existing/proposed manufacturing work station/workplace, and its associated tasks.

However, a number of previous researches have shown that most methods were focusing on the maintainability design or maintenance operation simulation by using VR. As a comprehensive parameter for describing maintainability design, maintenance time is mainly measured by maintenance experiments based on physical prototypes and relevant researches that use VR to study it is rare.

In a VM environment, inaccuracies in virtual peripherals and not considering the influence factors cause the simulation time of the maintenance process differing from actual process. A non-immersive, VM process consists of a series of virtual human action (Salvendy 1982). However, the question is how to use human action to measure action time, and what the inherent law is between human action and time.

The difference between maintenance process and line production should also be considered. The maintenance time is greatly influenced by the maintainability of the product. The method used in the line production is Method time Measurement (MTM) (Laring et al. 2002), which should be corrected in the maintenance time measurement. MTM is put forward by B.S.Q. Elzendoorn and Delphine Keller. A lot of literatures discussed MTM. In 1928, A. B. Segur raised the Motion-Time Mechanism in his Ph.D. thesis (Adams and Shoemaker 1989). Within actual conditions, he found that the time required for skilled personnel to finish a certain basic action is constant. This basic scientific theory has developed into a widely used method called the Predetermined Time Standard (PTS), which is also the theoretical basis for existing measurement of non-immersive, virtual human action time. Based on this idea, European and U.S. companies invented many PTS method until recent years, such as MTA (Motion time Analysis), WF (Work Factor System), MTM (Method time Measurement) and so on. In 1966, Dr. Heyde (G.C. Heyde) founded MOD method (Modular Arrangement of Predetermined Time Standard, MOD method for short) based on his long-term research (Ma et al. 2010). It is a most summary new method of PTS technology which combined time with action. MOD method is easy to learn and use, and it is convenient and practical to apply in engineering item. What’s more, its precision is not lower than the tradition PTS techniques.

However, the maintainability of the product will affect the use of MTM in measuring maintenance time. PTS is only appropriate in measuring the action of skilled workers in line production. Compared with actions, maintenance actions are more complex and accurate. How to apply PTS method in maintenance work considering these impact factors is still remained to solve. The MTM method is based on highest accuracy of nature and condition of gymnastic exercises. There is no double that this method would increase the difficulty of measuring, while the MOD method is relatively easy, the precision is also high. The MOD method is developed initially for formulating standard labor time, which is usually applied in product line, while the action in product line is different from the action in maintenance action, The operation in product lined is relatively easy and usually repetitive and the operator isn’t influenced by many factors, while the maintenance process is complex, for example sometimes the maintenance personnel have to use maintenance tools to operate. And the condition of the maintenance personnel is sometimes hard and influenced by visibility, maintenance space, accessibility, human factors. Because the maintenance simulation process isn’t showing the adjustment of the maintenance action caused by these factors. To predict the actual maintenance time, first we should modifies the MOD method considering the special action of the maintenance, second we should consider the influence factors to modifies the maintenance time to show up the time of adjustment. In this paper, the maintenance space, visibility and human posture are considered to correct the MOD method.

This paper is organized as follows. The “Framework of the proposed methodology” section provides the structure of the approach. In the “Time measurement of maintenance task”, a proper method of breaking down maintenance task and a reasonable virtual maintenance time measurement are presented. In section “Case study” a case is shown to verify the effectiveness the method, in section “Conclusion”, the conclusion and a few discussions are made.

## Framework of the proposed methodology

**Fig. 1:**
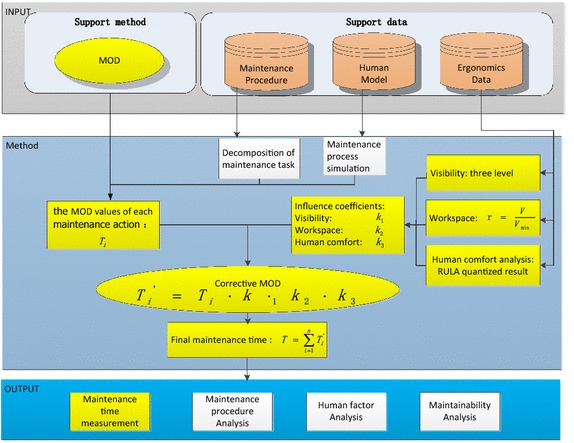
Frame work of the proposed methodology

Figure 1 show the framework which is constructed by four parts: support data, support method, method and output. The description of each part is as follows:

Support data is the data for research which are maintenance procedure, human model and ergonomics data. Maintenance procedure and human model are the basis to decompose the maintenance task into therbligs. Ergonomics data is used to study several impact factors which lead to the difference of the maintenance time between the actual maintenance work and simulation process.

Support method, in reference to MOD method, provides the key way to measure maintenance time of each therblig, and the method provides the way to study influence coefficients and maintenance time measure method, which will be discussed in detail in the following section.

Output introduces the output of the proposed work.

## Time measurement of maintenance task

Measuring maintenance time by MOD method regards human action as the core, so the decomposition of the maintenance task is the first step (Adams and Shoemaker 1989). Then maintenance personnel can portray maintenance actions particularly in virtual simulation environment and judges the MOD values of maintenance therbligs. Considering the influence of visibility, workspace and human posture, specific MOD value should be corrected. The final maintenance time can be got by adding up all maintenance MOD values.

### Decomposition of the maintenance task

Maintenance process should be decomposed before measuring maintenance time. Only composing all operation, then we can calculate the MOD of each therbligs to get the maintenance time of a maintenance event.

Generally, a maintenance process can be broken down into three levels: maintenance event, maintenance work and basic maintenance operation.

In the decomposition of the maintenance process, the basic maintenance action is the lowest level. The accuracy of prediction results are mainly determined by the accuracy of the basic maintenance action. However the results are mainly determined according to the experience and engineering practice, and lack of scientific and effective measurement method, which make the eventual prediction results unpersuasive. The basic maintenance operation is directly related to the product. For example, turning the screw, the process is different for the different product. This makes maintenance time of the same basic maintenance action different, and produces great influence on the final results. So it is necessary to study the basic maintenance time measurement to reduce artificial influences and improve the precision of the final prediction results.

**Fig. 2:**
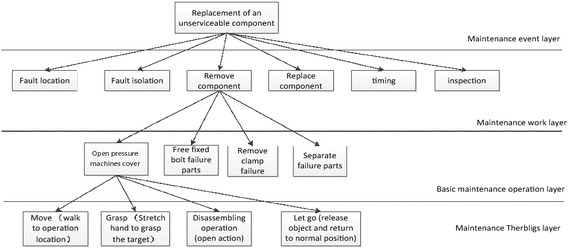
The hierarchical decomposition figure of a maintenance process

Human’s operation action can be divided into therbligs, so a maintenance process can be regard as a series of maintenance therbligs. Then maintenance work time measurement can be transformed into two aspects:

- Time measurement of the maintenance therbligs: Determine the basic time of maintenance therbligs. It has no relation to the product and has a certain generality.
- Layer design based on maintenance process: decomposing the maintenance process according to layering thought, bottom-up building, then the maintenance process can be decomposed into a series of sequential therbligs. Then, we add the maintenance therbligs layer into the traditional decomposition of the maintenance process. Finally we can decompose maintenance process into 4 layers: maintenance event layer, maintenance work layer, basic maintenance operation layer and maintenance therbligs layer. A typical maintenance process decomposition case is shown as Fig. 2.

As we can see from the Fig. 2, a maintenance operation contains a lot of maintenance action. Oriented to the maintenance task, this paper divides maintenance work into four parts considering characteristics of MOD method: mobile therbligs, posture-adjustive therbligs and operational therbligs.

### Corrective MOD

The basic principle of the MOD method derived from a large number of ergonomics experiments, which is summarized as follows:

- All operation actions of all personnel contain some basic movements. And the MOD method divides actual operation into 21 basic actions through a lot of experimental research;
- Different people consume the same time do if they do the same action under the same time condition.
- The time value of body movements of different parts is proportional to each other, for example the movement time of the hand is twice as much as finger gestures, and the forearm movement is 3 times as much as finger movement time. One finger motion time can be defined as the basic unit of human motion time; the time of other actions can be calculation by the multiple relationships with a finger action time.

According to the person’s action level, MOD method chooses the time consumption level of a finger gesture as the lowest action level, the fastest speed and the lowest energy consumption, as the unit time, which is remembered as 1 MOD. It means that finger moves, the average action time is 0.129 s, that is to say 1 MOD = 0.129 s (Ma et al. 2010). However, this conversion relation is not absolute, because different industries are not the same. In actual condition, the MOD time value can be determined according to the actual situation (Sun et al. 2009). MOD method divides human action into 21 categories including 11 basic therbilgs and 10 other auxiliary therbligs. 11 basic therbligs contain 5 mobile therbligs and 6 end therbligs. Mobiles therbligs mainly indicate that the position space of an object is changed by using fingers, wrists and arms;

End therbligs generally occurred after mobile therbligs, including scraping and place. Specific definitions and symbols of each therbligs are shown in Table 1 (Dong et al. 2008). It is convenient to use MOD method to calculate time. The MOD time value of a motion can be immediately got if we know the type of the movement. And the definition, symbols and classification of them are shown in Table 2.

**Table 1:** The MOD method classification of basic therbligs

Classification	Details	Sign
Move action		
Move	Finger movement	M1
	Wrist movement	M2
	Forearm movement	M3
	Upper arm movement	M4
	Straighten the arm movement	M5
Reflex motion	Continuous repeatedly reflex action	M1/2, M1, M2
End up action		
Grasping motion	Touch or contact	G0
	Grab without attention	G1
	Complicated capture	G3
Putting motion	Simple placement	P0
	Complex placement, as alignment	P2
	Placement aimed at assemble	P5
Other action		
Feet motion	Cadence motion	F3
Thigh motion	Walk action	W5
Independent motion (other actions have to stop)	Visual inspection	E2
	Correction	R2
	Judgment and reaction	D3
	Press down	A4
Body movements at the same time	Spins	C4
	Stoop/curve body → Stand up	B17
	Sit down → Get up	S30
Additive factor	Gravimetric factor (Load)	L1

**Table 2:** Add symbols and definitions

Definition	Sign	Details	Samples
Delayed	BD	one hand have movements, another hand is in the stopped state, don’t consume time	Right hand M Left hand BD
Maintain	H	Fixed state with hand holding or grasping objects, mainly refers to the action of support and fixed, don’t give time	Left hand H Right hand P2
Effective time	UT	Except movement of serviceman, inherent additional time caused by machines or other technical requirements instead of action produced, need to measure time accurately. Such as mechanical working time, soldering, riveting, testing, coating, etc.	Tin soldering time UT or Instrument testing time

#### Visibility factor analysis

Visibility is one of the most important influence factors in the maintenance work. It is defined as the degree of a region from one or more locations. In the maintenance category, visibility refers to the maintenance area within the scope of the sight, and good visibility can make maintenance personnel convenient to work. Visibility level directly affects the difficulty level of maintenance operation (Price 1991). Different from the line production task, limited to the design level, a lot of maintenance operation area has poor visibility in maintenance task, which makes maintenance personnel can’t operate normally and quickly (Heikkila et al. 2004). To complete the task as line production motion worker must go constant adjustment as a consequence, thus the maintenance time is extended. Ergonomics shows that the degree of visibility can be divided into three levels through large amount of experimental data:

1. The maintenance area is within the 15° scope cone, that is the best vision field;
2. The maintenance area is within the 30° scope cone, is the largest vision field;
3. The maintenance area is within the 30° scope cone, is the invisible field.

**Fig. 3:**
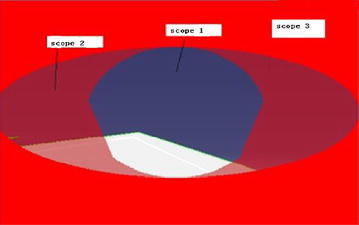
Vision scope

The virtual simulation software DELMIA support the visibility analysis tools, which can show us the detail information of scope cone, as shown in Fig. 3 (Chedmail et al. 2011).

Visibility evaluation criteria as shown in Table 3 (Briand et al. 1993). Assuming the distance between maintenance personnel’s eyes and maintenance operation object is h, the area of the best vision field, the largest vision field and the invisible maintenance operation is in the best vision, field.

**Table 3:** Visibility evaluation criteria

No	Visibility description	Scope analysis area	Visibility level
1	Maintenance objects and the operation can be directly seen during the whole maintenance process	15° scope cone	Good
2	Before maintenance activities, can see maintenance objects directly, but the maintenance operation is invisible due to physical or maintenance equipment/tools block when maintenance personnel is operating	35° scope cone	Normal
3	Maintenance objects and internal operations are not directly seen in the entire maintenance process, it is mainly depend on the experience, feeling and skill level	Other scope cone	Bad

Visibility doesn’t produce any hindering factors to maintenance operation. So it can be believed that there is no difference between the maintenance, operation and line production.

$$ \begin{aligned} S_{1} & = \pi \cdot (h \cdot \tan 15^\circ )^{2} \\ S_{2} & = \pi \cdot (h \cdot \tan 15^\circ ) \cdot (h \cdot \tan 35^\circ ) \\ \end{aligned} $$

The visibility influence coefficient is k_11_ = 1. When the visibility of the maintenance object is in the normal level, the maintenance personnel need to adjust his angel of view constantly to operate. Maintenance personnel operate in an environment which is complex. Sufficient operating room has to be reserved to avoid collisions during maintenance process (Ishii and Sato 1993). When the visibility level is in the normal level, the coefficient is k_12_ = 1.3. When the maintenance object is invisible, the coefficient is uncertain, time. For example: turning the screw, if there is not enough space. The maintenance time obeys the exponential distribution, and the maintenance time can’t be predicted. This design should be avoided in the maintenance design, the coefficient is k_13_ = 1.5. When we separately consider the impact of visibility, the MOD value of maintenance need to multiply the influence coefficient and the final MOD value can be obtained. The visibility influence coefficient result is shown in Table 4 (Seger 1987).

**Table 4:** Visibility influence coefficient

No	Visibility description	Scope analysis area	Visibility level	Influence coefficient
1	Maintenance objects and internal operations can directly be seen during the whole maintenance process	15° scope cone	Good	1
2	Before maintenance activities, can see maintenance objects directly, but the maintenance operation is invisible due to physical or maintenance equipment/tools block	35° scope cone	Normal	1.3
3	Maintenance objects and internal operations are not directly seen the entire maintenance process, mainly depend on the experience, feeling and skill level	Other scope cone	Bad	1.5

#### Workspace factor analysis

Workspace is defined as the actual space for maintenance personnel to work, which is an important factor for maintainability. When there is not enough workspace for the maintenance personnel, the maintenance personnel will consume more time to complete the maintenance task (Sato and Sakane 2000). Maintenance personnel need to constantly adjust their maintenance operation, therefore, the operation time will be prolonged. Here we define the basic maintenance actions are: screw, twist, translate in the Fig. 6, the maintenance space influence the basic operating we assume we rotate once, the angle is 10°, but if we rotate once, the angle is 20°, we will fix the screw in the half time. In evaluating the maintenance space level, scholars generally judge workspace by collision and interference (Duffy et al. 1989). And quantitative evaluation can be assessed by workspace ratio. When operation position and maintenance tools are determined, the minimum workspace can be calculated, the symbol is $V_{\rm min}$. And if the maintenance object is fixed, the maintenance space can also be gained. The workspace ratio is described as (Huang and Gupta 2005):

$$ r = \frac{V}{{V_{\rm min} }} $$

**Fig. 4:**
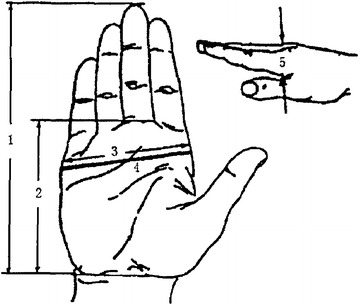
The schematic diagram of hand sizes

Maintenance operation is mainly divided into two kinds: bare-hand operation and using tools to operate (Abdel-Malek et al. 2004). Different kind has different way to calculate. The sizes of the maintainer’s hand are obtained through statistic data or related standards. Table 5 shows the different sizes of people’s hands in percentiles, and Fig. 4 shows the schematic diagram of the sizes (Zhou et al. 2014), which is got by using anthropometric techniques of Chinese population.

**Table 5:** The size of people’s hand in 5th pc, 50th pc, 95th pc (mm)

Items	Sex					
	Male			Female		
	5th	50th	95th	5th	50th	95th
1. Hand length	173	184	197	165	175	186
2. Palm length	99	105	112	93	99	105
3. Hand width	76	83	89	71	77	83
4. Palm perimeter	190	205	217	180	185	189
5. Palm thickness	27	28	30	24	25	26

**Fig. 5:**
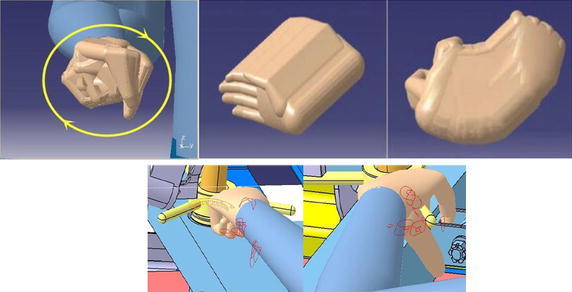
Workspace tool in DELMIA

According to Table 5 and Fig. 5 (Cutler et al. 1997), bare-hand minimum workspace can be set as a cube model. Length (defined as l) is the sum of hand length (named as $I_{\text{h}}$) and palm length (named as $I_{\text{p}}$); width (defined as w) is equal to the hand width (named as $w_{\text{h}}$); height (defined as h) is three times as hand thickness (named as h_t_).

So the bare-hand operation minimum workspace calculation expression is:

$$ V_{\text{min} } = l \cdot w \cdot h = 3(l_{h} + l_{p} ) \cdot w_{h} \cdot h_{t} $$

Assuming maintenance personnel use standard tools to operate the failed units. The size data of the standard tools can be got through relative industry standard. The with tool operation minimum workspace expression is (Lin et al. 2005)

$$ V_{\text{min} } = l_{t} \cdot \hbox{max} (w_{t} ,w_{h} ) \cdot \hbox{max} (h_{t}^{t} ,3h_{t} ) $$

where $l_{t}$ is the tool’s length; $w_{t}$ is the tool’s width; $h_{t}^{t}$ is the thickness of the tool.

By researching ergonomics data (An et al. 2007), the influence of workspace to the maintenance time can be divided into three levels. The detail of workspace evaluation criterion and influence coefficient is shown in Table 6. As a result, the influence coefficient of the workspace factor is: k21 = 1.1, k22 = 1.2, k23 = 2. And the specific data can be gained by virtual simulation software DELMIA. Through simulating therbligs which is decomposed from maintenance task, we can get the workspace data, as shown in Fig. 5.

**Table 6:** Workspace evaluation criterion and influence coefficient

No	Workspace description	r	Workspace level	Influence coefficient
1	Arm and tools of the maintenance personnel have enough workspace in the natural condition, is convenient to complete maintenance operations	>1.8	Good	1
2	Maintenance personnel’s arm and tool seldom collide the surrounding equipment, adjusting within a certain permitted scope, is convenient to complete maintenance operations	1.5 < r < 1.8	Normal	1.1
3	Arm and tool still cannot avoid colliding the surrounding equipment within a certain permitted scope, is difficult to finish the maintenance operation	<1.5	Bad	2

#### Human posture factor analysis

Human posture comfort analysis plays an important role in maintenance operation evaluation. Human posture analysis considers the comfort level and fatigue level of maintenance personnel during maintenance process. Bad working posture will not only cause injury to muscles and skeletons but also lead to physiological fatigue which contributes to a decline in mental performance (Shikdar and Sawaqed 2004). Sanjog et al. (2015) has made an attempt to assess the contributing role of workstation design, working posture concerning symptoms of musculoskeletal ailments and to find out inter-relationship between these factors in manufacturing. Thus, the comfort level of human working posture has a great influence on work efficiency and maintenance time. In DELMIA, ergonomics analysis tool support RULA (rapid upper limb analysis) method (Mcatamney and Corlett 1993).

**Fig. 6:**
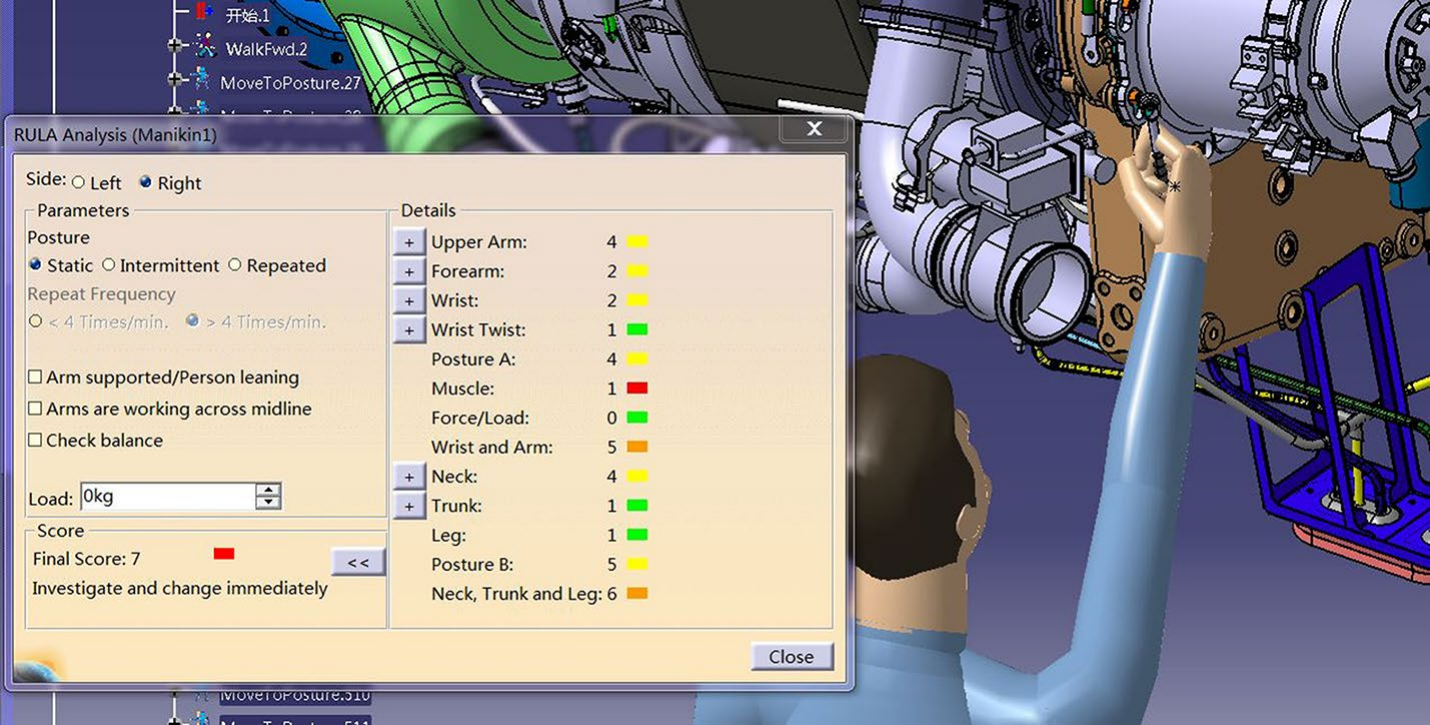
RULA analysis in DELMIA

**Fig. 7:**
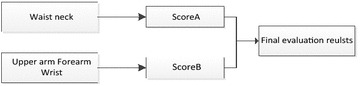
RULA evaluation process

As is shown in Fig. 6, it can evaluate the comfort level of the human posture and give maintenance personnel quantitative result. Detailed information of RULA method can be seen in Table 7 (Chaffin 1973; Kadefors 1980) From Table 7, green indicates that human posture is comfortable, and this posture can be received; yellow means that human posture is a little uncomfortable to some extent, but this posture is still in the received scope; brown state that the comfort level of this posture is poor, needs improving quickly; red explains maintenance personnel keeps a very bad working posture, posture needs changing right now to ensure maintenance safety (Hagberg 1981). RULA method divides these limb results into two groups, and analysis these result integrated and comprehensively. Detail analysis process is shown in Fig. 7 and Tables 8, 9 and 10 (Lindeman and Templeman 2001) Dr. Lynn McAtamney and Dr. E Nigel Corlett (the authors of RULA) finally divided the comfort quantitative result into five parts and gave some suggestion. According to the ergonomics data and experts’ experience, we can get the influence coefficient. Table 11 presents the influence coefficient of human posture factors. Dr. Lynn McAtamney and Dr. E Nigel Corlett (the authors of RULA) finally divided the comfort quantitative result into five parts and gave some suggestion.

**Table 7:** Detail information of RULA

Limb	Result range	Color relative to result					
		1	2	3	4	5	6
Upper arm	1–6	Green	Green	Yellow	Yellow	Red	Red
Forearm	1–3	Green	Yellow	Red			
Wrist	1–4	Green	Yellow	Brown	Red		
Wrist twist	1–2	Green	Red				
Neck	1–6	Green	Green	Yellow	Yellow	Red	Red
Body	1–6	Green	Green	Yellow	Yellow	Red	Red

**Table 8:** Table into which the l posture scores for the upper limbs are entered to find posture score A

Upper arm	Forearm	Wrist posture score							
		1		2		3		4	
		W	Twist	W	Twist	W	Twist	W	Twist
		1	2	1	2	1	2	1	2
1	1	1	2	2	2	2	3	3	3
	2	2	2	2	2	3	3	3	3
	3	2	3	3	3	3	3	4	4
2	1	2	3	3	3	3	4	4	4
	2	3	3	3	3	3	4	4	4
	3	3	4	4	4	4	4	5	5
3	1	3	3	4	4	4	4	5	5
	2	3	4	4	4	4	4	5	5
	3	4	4	4	4	4	5	5	5
4	1	4	4	4	4	4	5	5	5
	2	4	4	4	4	4	5	5	5
	3	4	4	4	5	5	5	6	6
5	1	5	5	5	5	5	6	6	7
	2	5	6	6	6	6	7	7	7
	3	6	6	6	7	7	7	7	8
6	1	7	7	7	7	7	8	8	9
	2	8	8	8	8	8	9	9	9
	3	9	9	9	9	9	9	9	9

**Table 9:** Table into which the individual posture scores for the neck, trunk and legs are entered to find posture score B

Neck posture score	Trunk posture score											
	1 Legs		2 Legs		3 Legs		4 Legs		5 Legs		6 Legs	
	1	2	1	2	1	2	1	2	1	2	1	2
1	1	3	2	3	3	4	5	5	6	6	7	7
2	2	3	2	3	4	5	5	5	6	7	7	7
3	3	3	4	4	4	5	5	6	6	7	7	7
4	5	5	5	6	6	7	7	7	7	7	8	8
5	7	7	7	7	7	8	8	8	8	8	8	8
6	8	8	8	8	8		8	9	9	9	9	9

**Table 10:** Final result

Final	Score B									
		1	2	3	4	5	6	7	8	9
Score A	1	1	1	1	2	3	3	4	5	6
	2	1	2	2	3	4	4	5	6	6
	3	2	3	3	3	4	5	6	7	7
	4	3	4	4	4	5	6	7	8	8
	5	4	4	4	5	6	7	8	8	9
	6	6	6	6	7	8	8	9	9	10
	7	7	7	7	8	9	9	9	10	10
	8	8	8	8	9	10	10	10	10	10
	9	9	9	9	10	10	10	11	11	11

**Table 11:** Human posture factor level and influence coefficient

Activity result	Final result	Risk level	Suggestion	Influence coefficient
0	1	No risk	No need to improve	1
1	2–3	Low	Maybe need to improve	1.24
2	4–6	Middle	Need to improve	1.37
3	7–9	High	Need to improve quickly	1.5
4	10–11	Polar high	Need to improve right now	1.63

The quantitative research on relation of work efficiency is rare during previous research, most of them only qualitatively describe the relation, but they can’t assure certain linear relation, we take example for human factor psychology There exits several hypothesis.

1. We assure the maintenance worker work in a steady state.
2. The relation between fatigue and work efficiency, so we determine the work efficiency is E, the rank of the fatigue is *E*_Normal_ F_rank_, when the maintenance worker is in the different condition.

The work efficiency is:

$$ {\text{E}} = \frac{{E_{\text{MAX}} - E_{\text{Normal}} }}{5} + E_{\text{Normal}} \cdot F_{\text{Rank}} $$

According to Jamieson (1990), the decline rate of the muscle is approximately 0.126, so the final result shows the influence coefficient of human posture is: k_30_ = 1, k_31_ = 1.24, k_32_ = 1.37 k_33_ = 1.5, k_34_ = 1.63.

#### Determine MOD value

As visibility, workspace and human posture have directly interactive relationship with these three factors influencing on maintenance time measurement, this paper assumes that visibility, workspace and human posture are three mutual independent factors in maintenance time measuring. Therefore, the MOD value can be calculated as following:

1. According to the proposed decomposition method, we can decompose the maintenance into therbligs. Maintenance personnel can simulate and decompose by virtual reality technology such as DELMIA;
2. Look up Tables 3, 6 and 11, find out the influence coefficients of visibility, workspace and human posture through ergonomics tools in DELMIA;
3. According to the basic therbligs, look up Table 1, determine the basic MOD value, and define it as $T_{i}$.
4. Weighting basic time of the ith maintenance therbligs with three influence coefficients comprehensively, gain the correct ith consuming time, define it as.
  $$ T_{i}^{\prime } = T_{i} \cdot k_{1} \times k_{2} \times k_{3} $$
5. Add all maintenance time of each therbligs, the final maintenance time can be calculated as $T = \sum\nolimits_{i = 1}^{n} {T_{i}^{\prime } }$.

## Case study

**Fig. 8:**
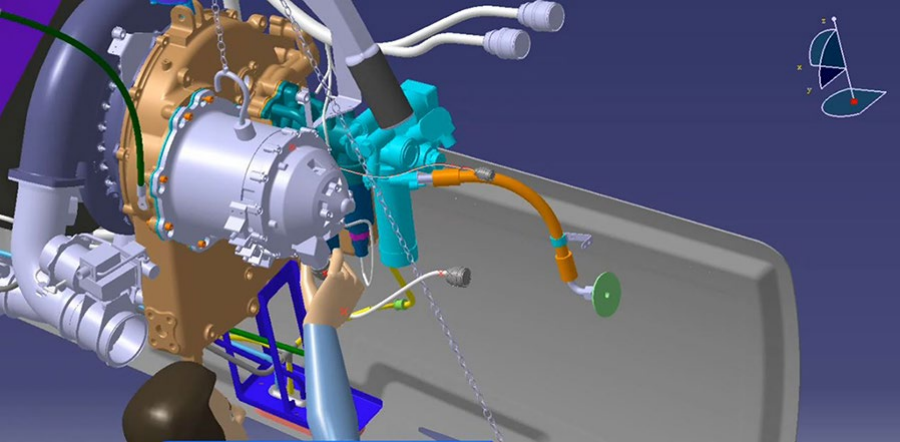
Location of the APU

The APU motor starter of an air bus is installed at the Empennage of the plane. It is fixed by 8 hexagon bolt on the bracket. The weight of APU is about 20 kg. Maintenance personnel have already built the virtual simulation maintenance environment in DELMIA. The location of APU is shown in Fig. 8.

### Decomposition of the maintenance process

**Fig. 9:**
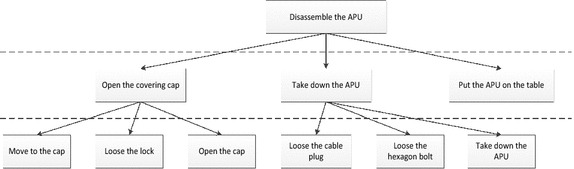
Decomposition of the maintenance process

According to the related chapters about APU motor starter in the maintenance manual, its maintenance process can be decomposed as Fig. 9.

### Establishment of the simulation model

**Fig. 10:**
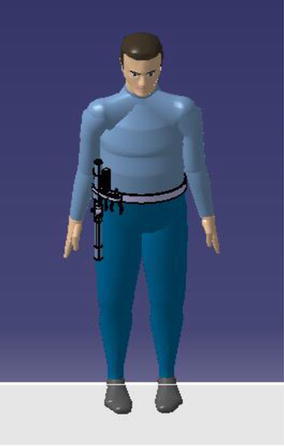
Virtual human model

Single maintenance personnel can finish disassembling the APU motor starter with only hexagon wrench. According to the human data in GB-10000-1988, we build a Chinese maintenance personnel model in DELMIA. As shown in Fig. 10.

**Fig. 11:**
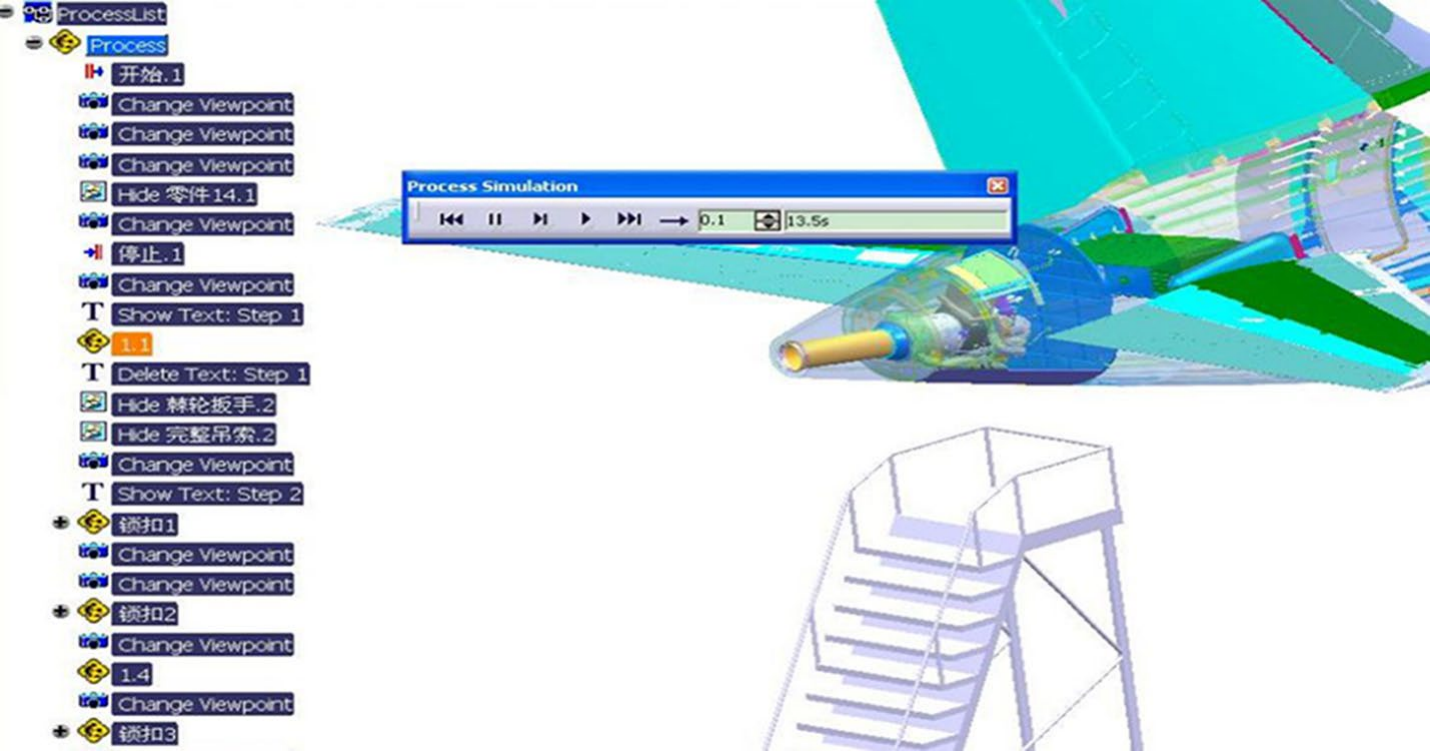
Layering structure tree of maintenance process

On the basis of APU decomposition of the maintenance process the layering structure tree is constructed under the ProcessList in DELMIA. As shown in Fig. 11. Then we set up HumanTask which is corresponded to the basic maintenance work and establish the maintenance therblig, and finally establish the simulation process of the whole maintenance process.

### Maintenance therbligs time measurement

**Fig. 12:**
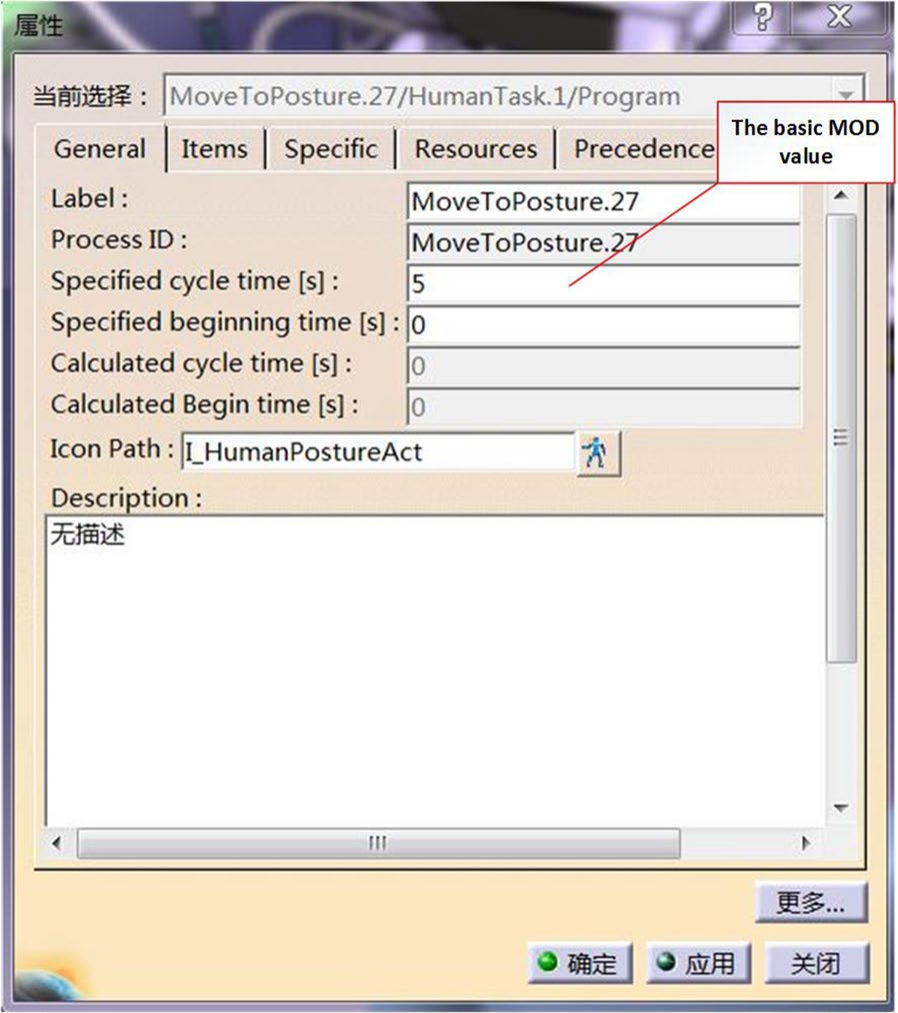
The property dialogue box

In DELMIA, a maintenance therbligs is named as “move to posture” (MTP). We can analyze its basic MOD value and fill the basic MOD value in the third blank space in the property dialogue box, as shown in Fig. 12.

**Fig. 13:**
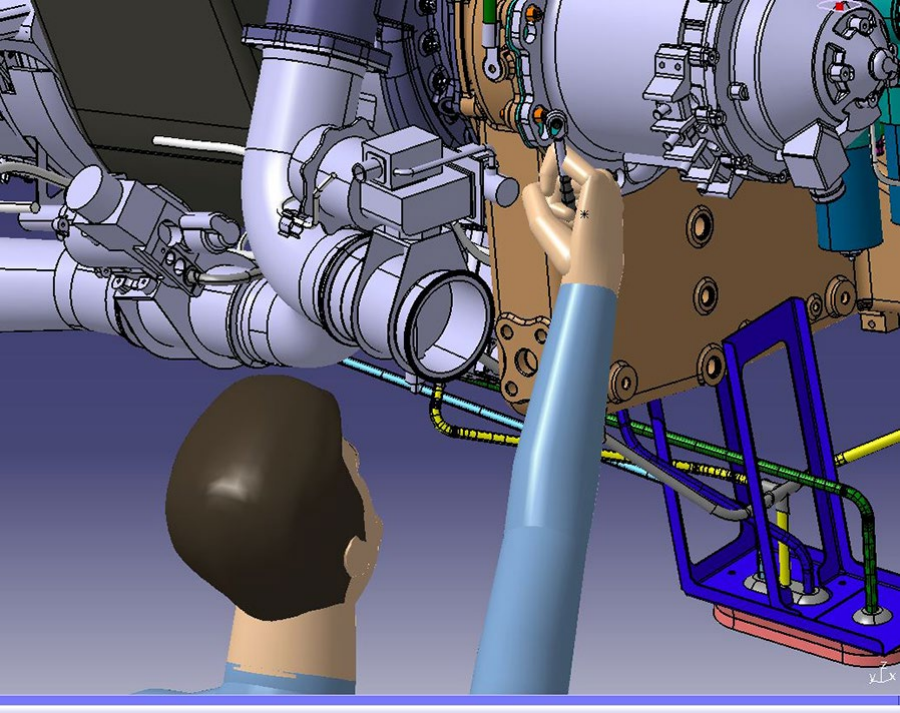
Maintenance personnel is screwing the hexagon bolt

Based on this method, the basic MOD value of each MTP can be filled in the property dialogue box. Then, we choose one human task open its Gantt figure, and we can acquire the under ProcessList in P.P.R environment, consumption MOD value of this human task in Fig. 13.

After we get the basic MOD value, the next step is correcting MOD value considering the influence coefficients of visibility, workspace and human posture. Taking a screw MTP as an example, the one of the maintenance therbligs is shown in Fig. 13.

In the figure, maintenance tool is ratchet wrench, its length is 200 mm, width is 30 mm and thickness is 15 mm. According to the content in “workspace factor analysis”, the minimum workspace is:

$$ V_{\rm min} = 200 \times 83 \times 3 \times 28 = 1.3944 \times 10^{6} ({\text{mm}}^{3} ) $$

And the workspace is:

$$ V = (260 \times 260 \times 300 - \pi 120^{2} \times 300)/4 = 1.6771 \times 10^{6} ({\text{mm}}^{3} ) $$

So the workspace ratio is:

$$ r = \frac{V}{{V_{\text{min} } }} = \frac{1.6771}{1.3944} = 1.203 < 1.5 $$

And the workspace influence coefficient is k_2_ = 1.3.

**Fig. 14:**
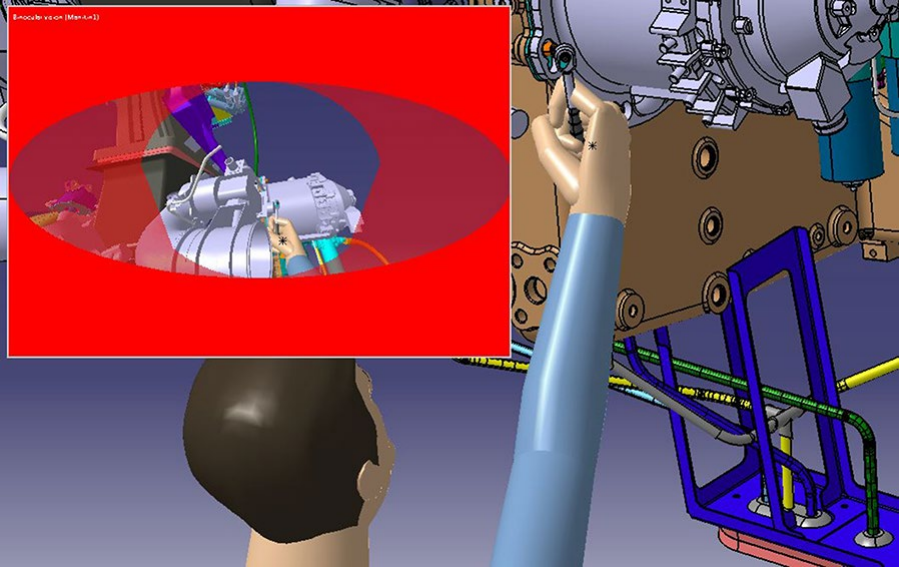
Visibility condition

**Fig. 15:**
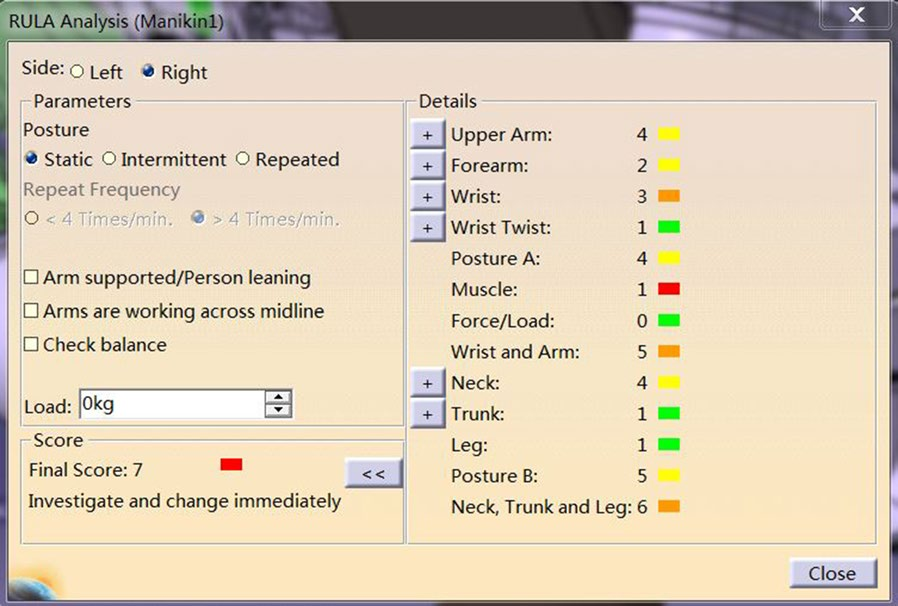
RULA analysis result

Opening the tool eye vision, the visibility condition under this MTP can be gained by Fig. 14. From Fig. 14, we find that maintenance personnel cannot see the target hexagon bolt. According to the content in “visibility factor analysis”, the visibility influence coefficient is k_1_ = 1.3. We open the human ergonomics tool and choose the RULA analysis tool; the result can be seen in Fig. 15. Look up to the contents in chapter “human posture analysis”, the final RULA quantitative result is 8, and the human posture influence coefficient is k_3_ = 1.5.

As the basic MOD value of this maintenance therbligs is belongs to M2, so the basic consumption time is 2 MOD. The corrective MOD time is 2 MOD. And the value is:

$$ T_{i}^{\prime } = T_{i} \cdot k_{1} \cdot k_{2} \cdot k_{3} = 2 \times 1.3 \times 1.5 \times 1.3 = 5\;MOD $$

The rest can be done in the same manner. Then we can measure all maintenance therbligs MOD values.

### The basic maintenance work time measurement

We can measure the basic maintenance work some therbligs. Therefore through cumulating the maintenance therbligs time, the total time can be gained. The procedure of disassembling APU motor starter can be divided into 8 parts: going up the operation platform, opening seven cap locks, opening the left side cabin door, opening the right side cabin door, screwing 8 hexagon bolts, screwing APU, moving cables and putting APU to the operation platform.

With the help of Gantt figures of the 8 basic maintenance works, the basic maintenance work measurement time and corrective time is presented in Table 12 in the below. From Table 12, we can see that maintenance time measured directly by MOD method is 202.659 s; while the maintenance time measured by corrected MOD method is 289.734 s, which is closer to the actual maintenance time 296.266 s than the previous supplement influence coefficients for correcting MOD method to obtain maintenance time. Finally, proposed methodology demonstrates the effectiveness and rationality of the methodology in supporting product maintainability design and time measurement in early stage of product design stage.

**Table 12:** Basic maintenance time measurement results and comparison of three groups

Num	Item name							
	Basic maintenance work	Basic MOD value	Basic consumption time/s	Proportion for actual time (%)	Corrective MOD value	Corrective consumption time/s	Proportion for actual time (%)	Actual consumption time/s
1	Going up the 788operation platform	595	76.755	90.542	639	82.431	97.237	84.773
2	Opening seven cap locks	235	30.315	80.599	282	36.378	96.719	37.612
3	Opening the left side cabin door	55	7.095	62.788	78	10.062	89.044	11.300
4	Opening the right side cabin door	55	7.095	70.5	78	10.062	89.044	11.300
5	Screwing 8 hexagon bolts	415	53.535	50.702	837	107.973	102.260	105.587
6	Screwing APU	18	2.322	61.920	25	3.225	86	3.750
7	Moving cables	49	6.321	59.380	74	9.546	89.676	10.465
8	Putting APU to the operation platform	149	19.221	61.028	233	30.057	95.483	31.479
Total		1571	202.659	68.404	2246	289.734	97.795	296.266

## Conclusion

Maintainability is product’s ability to maintain and restore condition according to prescribed procedures and methods to repair under the stated conditions for a given period of time. The prescribed time limit to complete maintenance work is metric to measure good degree of product’s maintainability, which influences the availability of the product directly and influences the combat readiness. For example, in military fields, good maintainability will reduce the downtime caused by maintenance, improve the executing ability of equipment to increase combat efficiency, and also in the field of manufacturing, downtime of product line caused by maintenance will cause great loss, but now there isn’t a useful method to measure the maintenance time in the design stage to verify if the maintenance time can meet maintainability requirement, while traditional way rely on statistic data in the physical prototypes in the later stage, which is lagged, and modifies the design is very difficult. To predict actual maintenance time in the design phase, this paper presents a method for measuring the actual maintenance time through virtual maintenance process simulation for complex product system. Considering unique features of the maintenance, this paper proposes a corrective MOD method to measure time, because VR still exist difference from the reality, it is difficult to simulate the adjustment process of the maintenance personnel, considering the some influence factors such as workspace, visibility and human posture that influence the maintenance operation, this paper modifies the initial maintenance time of each therbligs got from the virtual simulation. Finally a case verifies the effectiveness of the proposed method .

## Abbreviations

MOD: modular arrangement of predetermine time system
MTM: method–time–measurement
